# Proanthocyanidins Modulate MicroRNA Expression in Human HepG2 Cells

**DOI:** 10.1371/journal.pone.0025982

**Published:** 2011-10-05

**Authors:** Anna Arola-Arnal, Cinta Bladé

**Affiliations:** 1 Department of Biochemistry and Biotechnology, Universitat Rovira i Virgili, Tarragona, Spain; 2 Centre Tecnològic de Nutrició i Salut (CTNS), Reus, Spain; Florida State University, United States of America

## Abstract

Mi(cro)RNAs are small non-coding RNAs of 18-25 nucleotides in length that modulate gene expression at the post-transcriptional level. These RNAs have been shown to be involved in a several biological processes, human diseases and metabolic disorders. Proanthocyanidins, which are the most abundant polyphenol class in the human diet, have positive health effects on a variety of metabolic disorders such as inflammation, obesity, diabetes and insulin resistance. The present study aimed to evaluate whether proanthocyanidin-rich natural extracts modulate miRNA expression. Using microarray analysis and Q-PCR, we investigated miRNA expression in HepG2 cells treated with proanthocyanidins. Our results showed that when HepG2 cells were treated with grape seed proanthocyanidin extract (GSPE), cocoa proanthocyanidin extract (CPE) or pure epigallocatechin gallate isolated from green tea (EGCG), fifteen, six and five differentially expressed miRNAs, respectively, were identified out of 904 mRNAs. Specifically, miR-30b* was downregulated by the three treatments, and treatment with GSPE or CPE upregulated miR-1224-3p, miR-197 and miR-532-3p. Therefore, these results provide evidence of the capacity of dietary proanthocyanidins to influence microRNA expression, suggesting a new mechanism of action of proanthocyanidins.

## Introduction

MicroRNAs (miRNAs) are small non-coding RNAs of 18-25 nucleotides in length that bind to complementary 3′UTR regions of target mRNAs, inducing the degradation or transcriptional repression of the target [1]. A single miRNA can regulate the expression of multiple target mRNAs [2]. To date, more than 15000 miRNAs have been recorded in the miRBase database (www.mirbase.org/, April 2011), and it is thought that these tiny molecules may regulate approximately 30% of all cell transcripts [3], [4]. miRNAs have been reported to regulate several metabolic pathways such as insulin secretion, cholesterol biosynthesis and triglyceride, carbohydrate and lipid metabolism [5], [6], [7]. In addition, miRNAs have been shown to be involved in other biological processes such as differentiation and development [8]. Furthermore, not only have miRNAs be shown to be related to several human diseases, there is also evidence that the modulation of miRNAs can provide therapeutic benefits [9], [10], [11]. Interestingly, dietary factors, including micronutrients and non-nutrient dietary components, have been shown to alter miRNA gene expression [12]. For instance, dietary polyphenols such as soy isoflavones [13] and the green tea polyphenol epigallocatechin gallate [13], [14] have been shown to modulate miRNA expression.

More than 8000 phenolic structures have been reported in plants, and many of them occur in food [15]. Interestingly, the phenolic compositions of food plants differ considerably between sources, and several databases are emerging that provide quantitative information on the phenol content of foods. These databases include the USDA [16] and Phenol-Explorer databases. Proanthocyanidins are the most abundant polyphenols in the human diet, mainly provided by fruits, beans, nuts, cocoa, tea and wine [15], [17]. Proanthocyanidins have been shown to play important roles in several biological processes resulting in health benefits. For instance, proanthocyanidins have been reported to have antioxidant, anti-inflammatory, antimicrobial, antiproliferative, cardioprotective, hypolipidemic and antidiabetic activities [17], [18], [19].

Different proanthocyanidin extracts have been studied to determine their health and biochemical effects. Among them, grape seed proanthocyanidin extract (GSPE) and cocoa proanthocyanidin extract (CPE) have been used extensively. Proanthocyanidin extracts contain a broad range of different molecular structures characteristic of their botanical origin. Both GSPE and CPE can range in molecular weight from monomers to long-chain polymers. However, GSPE is mainly composed of trimeric proanthocyanidins [20], whereas CPE primarily contains dimeric proanthocyanidins [21]. Moreover, GSPE contains considerable amounts of galloylated monomers, such as epicatechin gallate and epigallocatechin gallate [20], whereas CPE contains little amounts of epigallocatechin [21]. Differences in the chemical structures of the proanthocyanidins present in the extract, such as the degree of polymerization and/or the presence of galloyl moieties, have been demonstrated to be important for proanthocyanidin functions [22], [23], [24]. For example, galloylated polyphenols have been shown to have greater inhibitory effects than non-galloylated polyphenols on pancreatic lipase [22].

Because polyphenols exhibit potent free radical-scavenging properties, it was thought that polyphenols have beneficial health effects by acting as antioxidants. However, it is now evident that proanthocyanidins modulate cell functionality by affecting intracellular signaling cascades and gene expression [15], [18]. As miRNAs have been reported to regulate several metabolic pathways [6], the objective of this study was to identify potential miRNA targets of proanthocyanidins in hepatocytes to identify new molecular mechanisms of action of these polyphenols. Proanthocyanidin extracts contain a broad range of different molecular structures characteristic of their botanical origin. For this reason, we used two different proanthocyanidin extracts (GSPE and CPE) and pure epigallocatechin gallate (EGCG) to determine which miRNA is the most universal target of proanthocyanidins. miR-1224-3p, miR-197 and miR-532-3p were differentially expressed after treatment with the two procyanidin extracts; however, only miR-30b* was differentially expressed in response to all the three treatments. Four hundred and eighty gene targets for miR-30b* have been validated, some of which are central to lipid and glucose metabolism, insulin signaling, oxidative stress and inflammation. All of these processes have been suggested to be responsible for the health effects of proanthocyanidin consumption.

## Results

### miRNA expression profiles in HepG2 cells treated with proanthocyanidin extracts or EGCG

To evaluate the capacity of proanthocyanidins to modify miRNA expression, all human miRNAs were screened in human hepatocellular carcinoma (HepG2) cells. HepG2 cells were treated for 5 h with 100 mg/L of grape seed or cacao proanthocyanidin extracts or with 50 mg/L of EGCG. GSPE and CPE were selected because they have different molecular compositions [20], [21] and are known to have beneficial health effects on metabolic disorders. Moreover, EGCG was selected because it is present in GSPE but not in CPE [20], [21]. Two biological samples for each treatment were analyzed by microarrays, and differentially expressed miRNAs were identified by using raw p value a filter based on statistical significance (*P*<0.05) as calculated by the Limma (Bioconductor) test due to the small sample size.

EGCG and the two procyanidin extracts modulated miRNA expression in HepG2 cells compared to the control cells. GSPE treatment (Table 1) differentially upregulated the expression of nine miRNAs and repressed the expression of six miRNA, whereas CPE treatment (Table 2) differentially repressed the expression of three miRNAs and upregulated the expression of three other miRNAs. Finally, EGCG treatment differentially repressed the expression of five miRNAs. These results indicated that the wide variety of molecular structures present in the extracts controlled the expression of a greater number of miRNAs compared to pure EGCG. Moreover, GSPE had a greater miRNA-modulation effect than CPE.

**Table 1:** miRNAs differentially expressed in HepG2 cells after 5 hours of culture with grape seed proanthocyanidin extract.

miRNA	Chromosome localization	Fold change	P Value
*upregulated*			
has-miR-1224-3p	*3q27.1*	2.67 (2.83 ± 0.29)	0.000
has-miR-449b*	*5q11.2*	1.83	0.004
has-miR-197	*1p13.3*	1.68	0.009
has-miR-1249	*22q13.31*	1.54	0.020
has-miR-1234	*8q24.3*	1.49	0.020
has-miR-532-3p	*Xp11.23*	1.28	0.045
has-miR-15b*	*3q25.33*	1.27	0.041
has-miR-522	*19q13.42*	1.25	0.045
has-miR-744*	*17p12*	1.24	0.049
*downregulated*			
has-miR-2110	*10q25.3*	−1.25	0.045
has-miR-483-5p	*11p15.5*	−1.29	0.043
has-miR-320c	*18q11.2*	−1.32	0.040
has-miR-453	*14q32.31*	−1.43	0.022
has-miR-1290	*1p36.13*	−1.45	0.021
has-miR-30b*	*8q24.22*	−2.01 (−1.14 ± 0.17)	0.002

The fold change value and the standard deviations obtained by QRT-PCR is presented in parentheses.

**Table 2:** miRNAs differentially expressed in HepG2 cells after 5 hours of culture with cocoa proanthocyanidin extract.

miRNA	Chromosome localization	Fold change	P Value
*upregulated*			
has-miR-197	*1p13.3*	1.48	0.016
has-miR-1224-3p	*3q27.1*	1.36 (3.59 ± 0.74)	0.028
has-miR-532-3p	*Xp11.23*	1.29	0.036
*downregulated*			
has-miR-765	*1q23.1*	−1.25	0.043
has-miR-187*	*18q12.2*	−1.23	0.046
has-miR-30b*	*8q24.22*	−1.48 (−0.18 ± 0.25)	0.016

The fold change value and the standard deviations obtained by QRT-PCR is presented in parentheses.

To validate the microarray results, two miRNAs (miR-1224-3p and miR-30b*) were selected for QRT-PCR quantification. Two biological samples for each treatment were analyzed by QRT-PCR. The selected miRNA expression levels obtained by Q-PCR were similar to those observed by microarray analysis (Tables 1, 2 and 3). Therefore, these results validated the microarray analysis.

**Table 3:** miRNAs differentially expressed in HepG2 cells after 5 hours of culture with epigallocatechin gallate.

miRNA	Chromosome localization	Fold change	P Value
*downregulated*			
has-miR-30b*	*8q24.22*	−1.56 (−1.03 ± 0.94)	0.037
has-miR-453	*14q32.31*	−1.33	0.041
has-miR-520-e	*19q13.42*	−1.33	0.038
has-miR-629	*15q23*	−1.25	0.045
has-miR-608	*10q24.31*	−1.88	0.046

The fold change value and the standard deviations obtained by QRT-PCR is presented in parentheses.

Interestingly, miR-30b* was repressed by all three treatments. No other overlaps were observed for EGCG and CPE, whereas both GSPE and EGCG treatments repressed miR-453. On the other hand, the two procyanidin extracts upregulated the expression of miR-1224-3p, miR-197 and miR-532-3p. These show that the cacao and grape seed proanthocyanidin extracts were more similar to each other than to EGCG.

### Identification of the target genes of the differentially expressed miRNAs using bioinformatics analysis

For further analysis of the results, bioinformatics studies were performed to identify the target genes of the differentially expressed miRNAs. We focused our attention on miR-30b* because it was the only miRNA affected by the three treatments. The miRWalk online database (http://www.umm.uni-heidelberg.de/apps/zmf/mirwalk/) contained 480 genes that have been experimentally verified as target genes of miR-30b*. In addition to having a role in cancer, proanthocyanidins have known health beneficial effects related to lipid and glucose metabolism, insulin signaling, oxidative stress and inflammation. Therefore, we focused our attention on miR-30b* target genes related to these pathways using the miRWalk, BioCarta and KEGG databases (Table 4). Interestingly, this bioinformatics investigation revealed validated target genes belonging to the following pathways: inflammation pathway (BioCarta), NK-kappa-B pathway (BioCarta), Peroxisome proliferators-activated receptors PPAR signaling pathway (KEGG), PPARA pathway (BioCarta), insulin pathway (BioCarta), insulin signaling pathway (KEGG), glycolysis and gluconeogenesis (KEGG), glycerolipid metabolism (KEGG), mitochondria pathway (BioCarta), oxidative phosphorylation (KEGG) and glutathione metabolism (KEGG).

**Table 4:** Selection of validated target genes for miR-30b* grouped by pathway.

Pathway Name(1)	Target gene(2)	Function of the encoded protein(3)
Inflammation	*TGFB1*	Member of the transforming growth factor beta (TGFB) family of cytokines.
	*PDGFA*	Member of the platelet-derived growth factor family.
	*IFNA1*	Interferon alpha 1.
NF-kappa-B	*RELA*	Component of the NF-kappa-B complex.
	*IKBKB*	I-kappa-B protein kinase B. Allows activation of the NF-kappa-B complex.
PPAR signaling	*FADS2*	Fatty acid desaturase. Desaturation of fatty acids.
	*LPL*	Lipoprotein lipase. Expressed in heart, muscle, and adipose tissue. Triglyceride hydrolase.
	*PDGFA*	Platelet-derived growth factor.
	*PTGS2*	Cyclooxygenase. Key enzyme in prostaglandin biosynthesis.
	*RELA*	Component of the NF-kappa-B complex.
	*JUN*	jun proto-oncogene.
	*HSPA1A*	70 kDa heat shock protein.
Insulin signaling	*JUN*	jun proto-oncogene.
	*PRKCI*	Protein kinase C iota.
	*PDE3A*	Cyclic nucleotide phosphodiesterase 3A. cGMP-inhibited.
	*IKBKB*	I-kappa-B protein kinase B. Allows activation of the NF-kappa-B complex.
	*GYS1*	Glycogen synthase.
	*SOCS1*	suppressor of cytokine signaling.
	*AKT1*	Serine-threonine protein kinase.
	*RHEB*	Small GTPase, RAS-related.
	*PTPRF*	Protein tyrosine phosphatase, receptor type.
Glycolysis and Gluconeogenesis	*GAPDH*	Glyceraldehyde-3-phosphate dehydrogenase.
	*PGM1*	Phosphoglucomutase 1.
Glycerolipid metabolism	*GPAM*	Glycerol-3-phosphate acyltransferase, mitochondrial. Catalyzes the initial and committing step in glycerolipid biosynthesis. Pivotal role in the regulation of cellular triacylglycerol and phospholipid levels.
	*LPL*	Lipoprotein lipase. Expressed in heart, muscle, and adipose tissue. Triglyceride hydrolase.
	*LCLAT1*	lysocardiolipin acyltransferase 1.
Mitochondria and Oxidative phosphorylation	*BCL2*	Integral outer mitochondrial membrane protein that blocks the apoptotic death of some cells.
	*ATP6V1C1*	Component of vacuolar ATPase (V-ATPase).
	*ATP6V1F*	Component of vacuolar ATPase (V-ATPase).
	*ATP6V0A1*	Component of vacuolar ATPase (V-ATPase).
	*COX8A*	Cytochrome c oxidase subunit VIIIA. Terminal enzyme of the respiratory chain.
Glutathione metabolism	*TXNDC12*	Thioredoxin. Roles in redox regulation, defense against oxidative stress, refolding of disulfide-containing proteins, and regulation of transcription factors.
	*IDH1*	Isocitrate dehydrogenase 1 (NADP+), found in the cytoplasm and peroxisomes.
	*GSTM4*	Glutathione S-transferase mu 4. Detoxification of electrophilic compounds, including carcinogens, therapeutic drugs, environmental toxins and products of oxidative stress, by conjugation with glutathione.
	*ANPEP*	Alanyl (membrane) aminopeptidase, Aminopeptidase N.

(1): from the KEGG (http://www.genome.jp/kegg/) and BioCarta (http://www.biocarta.com/) pathway databases.

(2): from the miRWalk database (http://www.umm.uni-heidelberg.de/apps/zmf/mirwalk/).

(3): from the KEGG (http://www.genome.jp/kegg/) and NCBI-Gene (http://www.ncbi.nlm.nih.gov) databases.

The miRWalk database also contains experimentally validated target genes for the additional three miRNAs (miR-1224-3p, miR-197 and miR-532-3p) that were differentially overexpressed in response to both proanthocyanidin extracts (Table 5), although the number of target genes for these miRNAs was much lower than for miR-30b*. The miRWalk database lists validated target genes independent of their organ and tissue. Thus, in the liver context in which we performed our studies, the significant validated genes for these miRNAs are related to transcription factors (RUNX3, MYC), proteolysis (BRCA1 and CAPNS1), inflammation (NFKB1 and RELB) and fatty acid synthesis (FASN).

**Table 5:** Validated target genes for the miRNAs differentially expressed in response to both grape seed and cocoa proanthocyanidin extracts.

miRNA	Target gene (1)	Function of the encoded protein(2)
has-miR-1224-3p	*DROSHA*	ribonuclease type III. RNA maturation.
	*AMELX*	
	*CALB1*	calbindin 1. Calcium metabolism.
	*EZH2*	member of the Polycomb-group (PcG) family.
	*PRNP*	prion protein.
	*ENAM*	
	*TNF*	proinflammatory cytokine that belongs to the tumor necrosis factor superfamily.
has-miR-197	*BRCA1*	Type of ubiquitin ligase (E3). Ubiquitin-mediated proteolysis.
	*CAPNS1*	Calpain, small subunit 1. Calcium-dependent cysteine proteases.
	*TUSC2*	
	*FUS*	component of the heterogeneous nuclear ribonucleoprotein (hnRNP) complex, involved in pre-mRNA splicing and the export of fully processed mRNA to the cytoplasm.
	*MYC*	Transcription factor. Plays a role in cell cycle progression, apoptosis and cellular transformation.
	*HMGA2*	non-histone chromosomal high mobility group (HMG) protein family member; these protein function as architectural factors and are essential components of the enhanceosome.
	*FASN*	fatty acid synthase. Catalyzes the synthesis of palmitate from acetyl-CoA.
	*TSPAN3*	cell-surface protein. Mediates signal transduction events that play a role in the regulation of cell development, activation, growth and motility.
	*IGF1*	insulin-like growth factor 1 (somatomedin C).
	*EFEMP2*	extracellular matrix protein.
	*IL6*	interleukin 6 (interferon, beta 2).
	*ACVR1*	activin A receptor.
	*NFKB1*	This gene encodes a 105 kD protein that can undergo cotranslational processing by the 26S proteasome to produce a 50 kD protein. The 105 kD protein is a Rel protein-specific transcription inhibitor, and the 50 kD protein is the DNA-binding subunit of the NF-kappa-B (NFKB) protein complex.
	*RELB*	Component of the NF-kappa-B complex.
has-miR-532-3p	*RUNX3*	Transcription factor.
	*CDKN2D*	cyclin-dependent kinase inhibitors.

(1): from the miRWalk database (http://www.umm.uni-heidelberg.de/apps/zmf/mirwalk/).

(2): from the KEGG (http://www.genome.jp/kegg/) and NCBI-Gene (http://www.ncbi.nlm.nih.gov) databases.

The estrogen receptor alpha (ESR1) was the only target gene validated for miR-453 (Table 6) that was differentially expressed in response to GSPE and EGCG.

**Table 6:** Validated target genes for the miRNAs differentially expressed in response to both grape seed proanthocyanidin extract and epigallocatechin gallate.

miRNA	Target gene(1)	Function of the encoded protein(2)
has-miR-453	*ESR1*	Estrogen receptor alpha.

(1): from the miRWalk database (http://www.umm.uni-heidelberg.de/apps/zmf/mirwalk/).

(2): from the KEGG (http://www.genome.jp/kegg/) and NCBI-Gene (http://www.ncbi.nlm.nih.gov) databases.

## Discussion

In this study, we investigated the effect of dietary proanthocyanidins on miRNA expression to identify potential miRNA targets of proanthocyanidins. To achieve this goal, we selected the HepG2 human hepatoma cell line because dietary proanthocyanidins have been reported to reach the liver at high levels [25]. Moreover, hepatocytes have the enzymatic machinery to metabolize these compounds (glucuronidation, sulfation and methylation). We used two proanthocyanidin extracts (GSPE and CPE) with different compositions in addition to pure EGCG to search for a universal proanthocyanidin target miRNA. This study was conducted shortly after treatment (five hours) because we were searching for miRNAs that are able to act as primary mediators that trigger the effects of proanthocyanidins on cell functionality and metabolism. Previous results have showed that *in vivo* changes in mRNA levels related to lipid metabolism occur after 5 hours of GSPE treatment [26].

As shown in Tables 1 and 2, proanthocyanidins induced up to 2.67-fold changes. These values were expected because food compounds, in contrast to drugs, normally induce low levels of gene expression changes. Interestingly, GSPE was more effective than CPE with respect to modulating miRNA expression, probably due to the different chemical compositions. This result suggests that each proanthocyanidin may influence particular miRNAs. Proanthocyanidins can have a variety of structures that include different (i) chain lengths (this is known as the degree of polymerization), (ii) hydroxylation patterns, (iii) stereochemistries at the three chiral centers and (iv) locations and types of interflavan linkage [27]. Furthermore, the characteristic composition of an extract varies based on its botanical origin, the growing conditions of the cultivar and processing [15]. Therefore, we hypothesized that a specific proanthocyanidin, or a specific interaction between compounds, may affect a miRNA specifically.

On the other hand, HepG2 treatment with the monomeric EGCG was less successful in modifying miRNA expression. Several studies have focused their attention on the effect of the degree of polymerization on the biological activity of proanthocyanidins. For instance, the oxidation-inhibiting power of polymerized oligomers is much stronger than that of monomers [28]. The monomeric and dimeric structures do not reproduce the bioactivity of GSPE in glucose and lipid metabolism and only act as anti-inflammatory agents [29]. In contrast, a trimeric-enriched fraction of GSPE reproduces all of the biological effects of GSPE [29]. Thus, EGCG could affect fewer miRNA because of its low polymerization degree. Moreover, EGCG has more similarities to GSPE than to CPE. Gallate derivatives of proanthocyanidins are more abundant in GSPE than in CPE [20], in which only traces of epigallocatechin, and not EGCG, have been found [21].

Other authors have studied the influence of EGCG on the expression levels of miRNAs in HepG2 cells [14]. However, there was no overlap between the miRNAs differentially expressed in response to EGCG in the work of Tsang and Kwok [14] and those in the present study. This discrepancy could be explained by the different periods of treatment and the different doses: our miRNA experiments were performed after 5 hours of treatment with a dose of 50 µM, whereas in [14], the treatment was performed for 24 hours with a dose of 100 µM.

Interestingly, miR-30b*, the sole miRNA affected by the two proanthocyanidin extracts and EGCG, is the miRNA among those differentially expressed in response to proanthocyanidins that has the greatest number of validated target genes. To our knowledge, no paper has been published regarding the specific role of has-miR-30b* in intracellular processes. On the other hand, 480 target genes of miR-30b* have been experimentally verified. These target genes are implicated in numerous cell pathways, ranging from cell signaling to metabolism. Proanthocyanidins exert health benefits by improving lipid metabolism [18], glucose metabolism and insulin sensitivity [30] and by reducing inflammation [31], [32] and oxidative stress [33]. The target genes of has-miR-30b* are included in pathways involved in these processes. Thus, miR-30b* likely mediates part of the beneficial health effects of proanthocyanidins.

Proanthocyanidins induce a strong hypotriglyceridemic effect in experimental animals [18], in part by inhibiting VLDL secretion by the liver [34], [35]. miR-122 and miR-33, among others, play major roles in regulating cholesterol and fatty acid homeostasis in the liver [36]; however, neither of these miRNAs was modulated by the proanthocyanidins used in these experiments. However, fatty acid synthase, which plays a central role in liver lipogenesis [37], is a validated target gene of miR-197, which was upregulated by both proanthocyanidin extracts. It has been reported that oligomeric forms of proanthocyanidins specifically control endogenous liver lipid production [29], in accordance with the fact that miR-197 was only modulated by the extracts.

As we indicated in the introduction, proanthocyanidin extracts are a complex combination of different molecular structures. Thus, several molecular mechanisms by which proanthocyanidins modulate cell functionally have been described. It has been demonstrated that grape seed procyanidin extract activates the insulin receptor and key targets of insulin signaling pathways [30]. In addition, it has been shown that procyanidins decrease plasma triglyceride levels by activating the nuclear receptor FXR, transiently upregulating the nuclear receptor SHP expression and subsequently repressing SREBP1 in the liver [34], [35]. Moreover, proanthocyanidins reduce inflammation by inhibiting the activation of NFkB [31]. Thus, the modulation of miRNA expression may be an additional molecular mechanism by which proanthocyanidins, the most abundant polyphenols in the human diet, could modulate cell functionality.

## Materials and Methods

### Reagents

EGCG ((-)-Epigallocatechin gallate) was purchased from Sigma-Aldrich (Madrid, Spain), and GSPE (Grape Seed Procyanidin Extract) was kindly provided by Les Dérives Résiniques et Terpéniques (Dax, France). This procyanidin extract contained monomeric (10.1%), dimeric (12.3%), trimeric (77.1%) and oligomeric (4–6 units) (less than 1%) procyanidins. CPE (cacao procyanidin Extract) contained monomeric (27.8%), dimeric (44.8%), trimeric (21.1%), tetrameric (5.3%), and oligomeric (5–8 units) (1%) procyanidins.

### Cells and cell culture

HepG2 human hepatocarcinoma cells were purchased from the American Type Culture Collection (*ATCC*, *LGC Promochen,* HB8065, Salisbury, United Kingdom). The cells were cultured in Dulbecco's Modified Eagle Medium (DMEM, Lonza, BE12-614, Barcelona, Spain) supplemented with 10% fetal bovine serum (Lonza, BE-14-802-F, Barcelona, Spain), 0.1 mM nonessential amino acids (Sigma, M7145, Madrid, Spain), 100 U/ml penicillin and 100 µg/ml streptomycin (Lonza, BE-17-602E, Barcelona, Spain), 2 mM glutamine (Lonza, BE-17-605E, Barcelona, Spain), and 25 mM 4-(2-Hydroxyethyl)piperazine-1-ethanesulfonic acid (HEPES, Sigma, H3375, Madrid, Spain) in a humidified incubator with 5% CO_2_ at 37°C.

### miRNA microarray analysis

HepG2 cells were treated with 50 mg/L of EGCG, 100 mg/L of GSPE or 100 mg/L of CPE dissolved in dimethyl sulfoxide (DMSO, D8418, Sigma, Madrid, Spain), (final DMSO concentration in growth media, 0.1%) for 5 h. For control samples HepG2 cells were treated with 0.1% DMSO in growth media. Total RNA was isolated using the miRNeasy Mini Kit (QIAGEN, Izasa, Barcelona, Spain) following the manufacturer's protocol. For this analysis, febit's “Geniom Biochip MPEA *homo sapiens*” biochip (febit biomed gmbh, Heidelberg, Germany) was used. The probes were designed as the reverse complements of all major mature miRNAs and the mature sequences as published in the current Sanger miRBase release (version 14.0 September 2009, http://microrna.sanger.ac.uk/sequences/index.shtml) for *Homo sapiens*. Additional nucleotides were added to the 5' end of each capture oligonucleotide as necessary for the enzymatic extension in the labeling procedure. The probes were synthesized with intra-array replicates to increase the statistical confidence and to compensate for potential positional effects. As a result, the raw data files contained 7 data points for each miRNA. The intensities of blank probes, which consisted only of one single “T” nucleotide, were used for background corrections. Spike-in controls were also performed for the labeling efficiency. To better control the hybridization process and the positioning features, additional hybridization controls were added to the array template. For each array, the RNA was suspended in febit's proprietary miRNA Hybridization Buffer (25* µl* per array, febit biomed gmbh, Heidelberg, Germany). Hybridization was performed automatically for 16 h at 42°C using a Geniom RT®-Analyzer (febit biomed gmbh, Heidelberg, Germany). In the next step, the biochip underwent a stringent wash. Following the labeling procedure, febit ran a microfluidic-based primer extension assay. This assay utilizes the bound miRNAs as a primer for an enzymatic elongation with labeled nucleotides. The elongation was performed with Klenow Fragments and biotinylated nucleotides at 37°C for 15 minutes. Finally, the biochip was washed automatically. For maximum sensitivity, febit used Biotin-11-dATP (PerkinElmer, Germany), which was detected with streptavidin-phycoerythrin (SAPE), in combination with febit's consecutive Signal Enhancement (CSE) procedure. The feature recognition (using Cy3 filter set) and signal calculation were performed automatically within milliseconds.

### Bioinformatics and statistical analysis

All bioinformatic and statistical analysis was provided by febit (febit biomed gmbh, Heidelberg, Germany). Briefly, spatial effects on the chip were investigated and corrected. Then, the intensity value distribution of the raw data was analyzed and, if required, normalized using variance stabilizing normalization (VSN). The Limma test using Empirical Bayes Statistics was used for the detection of differentially regulated miRNAs (raw p values < 0.05)

### miRNA target prediction

miRNA target genes were identified using the miRWalk online database (http://www.umm.uni-heidelberg.de/apps/zmf/mirwalk/). miRWalk provides information on published pathway targets from the KEGG (http://www.genome.jp/kegg/) and BioCarta (http://www.biocarta.com/) pathway databases. The functions of genes were obtained from KEGG and NCBI-Gene (http://www.ncbi.nlm.nih.gov).

### Quantitative real-time PCR (qRT-PCR)

HepG2 cells were treated with 50 mg/L of EGCG, 100 mg/L of GSPE or 100 mg/L of CPE dissolved in DMSO (D8418, Sigma, Madrid, Spain), (final DMSO concentration in growth media, 0.1%) for 5 h. For control samples HepG2 cells were treated with 0.1% DMSO in growth media. Total RNA was isolated using the miRNeasy Mini Kit (QIAGEN, Izasa, Barcelona, Spain) following the manufacturer's protocol. Two biological replicates were used for the analysis. Total RNA (5 ng) was reverse-transcribed using the TaqMan® MicroRNA Reverse Transcription kit (Applied Biosystems, Madrid, Spain) and the miRNA-specific reverse-transcription primers provided with the TaqMan® MicroRNA Assay (Applied Biosystems, Madrid, Spain). For the reverse-transcription, a My Gene L Series Peltier Thermal Cycler (Long Gene, Madrid, Spain) was used. The reaction was performed at 16°C for 30 min; 42°C for 30 min and 85°C for 5 min. The obtained miRNA-specific cDNA was amplified using the TaqMan Universal PCR master mix (Applied Biosystems, Madrid, Spain) and the respective specific probe provided in the TaqMan® MicroRNA Assay (Applied Biosystems, Madrid, Spain). The targeted miRNA assay sequences were 5′-CCCCACCUCCUCUCUCCUCAG-3′ for miR-1224-3p and 5′-CUGGGAGGUGGAUGUUUACUUC-3′ for miR-30b*. PCR was performed using a Real-Time 7300 PCR System (Applied Biosystems, Madrid, Spain). Amplification was performed at 95°C for 10 min, followed by 40 cycles of 95°C for 15 s and 60°C for 1 min. U6 small nuclear RNA was used as an endogenous control. The fold change in the miRNA level was calculated by the log 2 scale of the equation 2−ΔΔCt, where ΔCt  =  Ct miRNA-Ct U6 and ΔΔCt =  ΔCt treated samples- ΔCt untreated controls.
